# Association between Chronic Arsenic Exposure and Nutritional Status among the Women of Child Bearing Age: A Case-Control Study in Bangladesh

**DOI:** 10.3390/ijerph7072811

**Published:** 2010-07-02

**Authors:** Abul H. Milton, S. M. Shahidullah, Wayne Smith, Kazi S. Hossain, Ziaul Hasan, Kazi T. Ahmed

**Affiliations:** 1 Centre for Clinical Epidemiology & Biostatistics (CCEB), School of Medicine, The University of Newcastle, University Drive, Callaghan, NSW 2308, Australia; E-Mail: WSMIT@doh.health.nsw.gov.au (W.S.); 2 NGO Forum for Drinking Water Supply & Sanitation, 4/6, Block-E, Lalmatia, Dhaka-1207, Bangladesh; E-Mails: wqtl@ngof.org (S.M.S); ziahasan@hotmail.com (Z.H.); tanvir@ngof.org (K.T.A.); 3 Centre for Health and Development (CHAD), Ramdia, Bethuria, Kashiani, Gopalgonj, Bangladesh; E-Mail: h_d25@hotmail.com (K.S.H.)

**Keywords:** arsenic, nutritional status, reproductive aged women

## Abstract

The role of nutritional factors in arsenic metabolism and toxicity is yet to be fully elucidated. A low protein diet results in decreased excretion of DMA and increased tissue retention of arsenic in experimental studies. Malnourished women carry a higher risk of adverse pregnancy outcomes. Chronic exposure to high arsenic (>50 μg/L) through drinking water also increases the risk of adverse pregnancy outcomes. The synergistic effects (if any) of malnutrition and chronic arsenic exposure may worsen the adverse pregnancy outcomes. This population based case control study reports the association between chronic arsenic exposure and nutritional status among the rural women in Bangladesh. 348 cases (BMI < 18.5) and 360 controls (BMI 18.5–24.99) were recruited from a baseline survey conducted among 2,341 women. An excess risk for malnutrition was observed among the participants chronically exposed to higher concentrations of arsenic in drinking water after adjusting for potential confounders such as participant’s age, religion, education, monthly household income and history of oral contraceptive pills. Women exposed to arsenic >50 μg/L were at 1.9 times (Odds Ratio = 1.9, 95% CI = 1.1–3.6) increased risk of malnutrition compared to unexposed. The findings of this study suggest that chronic arsenic exposure is likely to contribute to poor nutritional status among women of 20–45 years.

## 1. Introduction

Arsenic contamination of drinking water is a public health problem in Bangladesh [1]. Extent of the drinking water arsenic contamination is unprecedented in rural areas of the country. Approximately one-third of the hand tube wells in Bangladesh contain arsenic more than 10 μg/L, the recommended level of arsenic in drinking water by the WHO [2]. Chronic exposure to arsenic has the potentials to cause wide ranges of carcinogenic and non-carcinogenic health effects such as cancer of the skin and internal organs, diabetes mellitus, hypertension, and respiratory conditions [3–10]. Among the non-carcinogenic effects, chronic arsenic exposure has also been reported to increase the risk of foetal and infant deaths [11–16].

Drinking water in Bangladesh contains mostly inorganic arsenic. Inorganic arsenic, once ingested is excreted through urine in human [17]. A series of reduction and oxidation methylation reactions occur in liver to form monomethylarsonic acid (MMA) and dimethylarsinic acid (DMA) [18]. Endogenous thiols probably play a critical role in the metabolic conversion of As (III) and As (V) species. Glutathion is likely to act as a reducing agent for converting As (V) species into As (III), which can then accept a methyl group from S-Adenosylmethionine (SAM) to produce the methyl arsenic (V) species [19]. Diet containing methionine, choline, folate, and other nutrients may be the source of this methyl group from SAM. However, the role of nutritional factors on arsenic metabolism and toxicity is yet to be fully explored.

Limited studies have indicated that poor nutritional status may increase the risk of arsenic related health effects [20–24]. Participants with poor nutritional status (weight below 80% of the standard body weight for their age and sex) were reported from West Bengal, India to have an overall 1.6 fold-increase (for males = 1.5, females = 2.1) in the prevalence of keratoses, suggesting that malnutrition may increase the susceptibility for arsenic toxicity [22]. Arsenic affected people of south western Taiwan and the Antofagasta region in northern Chile were reported to have a low socio-economic status and poor nutritional status [20,21,23,24]. Experimental studies have shown that rabbits fed diets containing low methionine, choline, or proteins have a decrease in arsenic methylation and an increase in tissue retention of arsenic, especially in the liver [25]. Decrease in urinary excretion of dimethyl arsinic acid (DMA) was also observed in another study where mice were fed a choline-deficient diet [26]. Lower Body mass index (BMI) was reported among the arsenicosis patients compared to the unexposed population in a previous study in Bangladesh [27]. In a recent study in Bangladesh, Heck *et al* observed that higher intakes of protein, methionine, and cysteine were associetd with 10–15% greater total urinary arsenic excretion, after adjusting for total energy intake, body weight, sex, age, tobacco use, and intake of some other nutrients [28]. However, a study from Atacameño in northern Chile reported no evidence of malnutrition among individuals with arsenic induced skin lesions [29]. Each of the families with arsenic-induced skin lesions consumed many vegetables, including carrots, everyday. This might be due to that high carotene intake and vitamin A do not prevent arsenic-induced skin lesions.

Malnutrition is highly prevalent in rural Bangladesh [27]. Malnourished women are likely to have higher risk of adverse pregnancy outcomes. Malnutrition during pregnancy may increase the risk of intra-uterine growth, low birth weight baby, stunting, severe wasting and foetal death [30,31]. Experimental studies on ewes and rats have shown that malnutrition during pregnancy resulted in modifications of embryo development leading to abnormalities of the offspring in late gestation and postnatal periods [32–34]. Chronic exposure to arsenic through drinking water also increases the risk of adverse pregnancy outcomes [11–16]. The combined effects of malnutrition and chronic arsenic exposure may worsen the adverse pregnancy outcomes. Therefore, the purpose of this case-control study was to determine the association between chronic arsenic exposure and nutritional status of the women of child bearing age in rural Bangladesh.

## 2. Methods

### 2.1. Study Area

The study was conducted in all the villages of the Nezamkandi union, Kashiani upazila (subdistrict) of the Gopalgonj district. The study area is a known arsenic contaminated area, although no individuals with visible arsenical skin lesions were observed from this area. The area is approximately 200 km south from Dhaka, the capital city of Bangladesh. The study was conducted during January 2006 to December 2008.

### 2.2. Selection of Cases and Controls

In this population based case-control study, 365 cases and 365 controls were recruited. A total of 335 cases and 335 controls with a case: control ratio of 1:1 are sufficient to demonstrate an odds ratio of 1.5 at a level of 95% confidence and 80% power in the study population where 30% of the controls are chronically exposed to arsenic concentration more than 50 μg/L. We recruited extra 30 cases and 30 controls to accommodate non-response, unavailability and refusal of the participants.

Initially a baseline survey was carried out among 2341 eligible women [35]. Eligible participants were women aged 20–45 years living in the study area for at least six months. Of these 2,341, 798 (34%) women were found as malnourished (BMI < 18.5). Currently pregnant women were not included in the baseline study. Three respondents were excluded from the analysis of baseline data as their interview schedules were incomplete. Three hundred sixty five cases and 365 controls were randomly selected from the sampling frame of cases (N = 798) and controls (N = 1,543). Women with a BMI < 18.5, aged between 20–45 years, have been drinking water from a tube well and have been a resident of the study area for the preceding six months were recruited as cases. Women having a BMI > 18.5 were recruited in the study as controls, other inclusion criteria for them were same as the cases. Pregnant women and women suffering from any chronic illness were excluded from the study. Of these cases and controls, 348 cases and 360 controls were included in the final analysis as rest of the participants was excluded for incomplete data. A total of 242 tube wells were used by the study participants. We obtained ethical approval from the Health Human Research Ethics Committee of The University of Newcastle, NSW, Australia and The Bangladesh Medical Research Council, Bangladesh.

### 2.3. Data Collection

A structured pretested interviewer-administered questionnaire was used to collect information from cases and controls on sociodemographic variables including household’s total monthly income, drinking water history, nutritional status and pregnancy related history. Six trained interviewers (three men and three women) administered the questionnaires by face-to-face interview. Standing height and weight of each participant were measured with the subjects wearing light clothes and not wearing shoes. Height was measured in centimetres using a locally made wooden measuring board with a metal measurement tape attached. Weight was measured in kilograms using bathroom scales that were calibrated daily and zeroed before each measurement. All measurements were made according to WHO procedures [36].

### 2.4. Outcome Definition

Individual nutritional status of the study women was assessed using body mass index (BMI), calculated as [weight (kg)]/[height (m)^2^]. BMI was interpreted as malnourished when <18.5, normal if ranging between 18.5 and 24.99 and overweighted if it exceeds 24.99 [37]. Cases and controls were those women had a BMI < 18.5 and BMI > 18.5, respectively.

### 2.5. Exposure Assessment

A single tube well water measurement was used to characterise chronic arsenic exposure for each study participant. We calculated duration of individual arsenic exposure from the period of use of the particular tube well. Water samples were collected, transferred and preserved following standard procedures [38]. All these water samples were analysed for arsenic at the NGO Forum for Drinking Water Supply and Sanitation’s water quality testing laboratory in Dhaka using flow-injection hydride generation atomic absorption spectrometry method [38]. The minimum detection level for arsenic is 3 μg/L in this method.

### 2.6. Statistical Analysis

Data analysis was carried out using Stata 9.0 statistical software (StataCorp LP, TX, USA). Baseline characteristics were compared among cases and controls using chi-square and t-test as appropriate.

Logistic regression analysis was done to calculate odds ratios (ORs) and 95% confidence intervals (CIs) for each outcome, initially adjusted for the potential confounders of participant’s age, education, religion, marital status, monthly total household income, type of roof, age at menstruation, duration of menstruation, age at marriage, history of oral contraceptives, and total number of pregnancy. In the final logistic regression models, we only included those variables found to be significant at 25% (p < 0.25) level in the initial models to accommodate more explanatory variables in the final model and to reduce type II error.

Arsenic concentration was categorised as ≤50 μg/L and >50 μg/L, (and subcategorised as) 51–100 and >100 μg/L; for each, duration of exposure was categorised as up to 10 years and more than 10 years. Confidence intervals from the final analysis for cases and controls were based on standard errors adjusted for clustering of outcomes among women using the same tube well using Huber/White robust ‘sandwich’ estimator of variance, which is based on the within-cluster correlation observed in the data.

## 3. Results and Discussion

Table 1 presents the baseline characteristics, arsenic exposure and nutritional status of the cases and controls. Cases are similar to the controls with respect to its baseline characteristics except total monthly household income, arsenic concentrations and body weight.

The combined effects of arsenic concentration and duration of arsenic exposure on nutritional status are presented in Table 3. For arsenic concentration of 100 μg/L with an exposure of ≤10 years, the risk estimate for malnutrition is high. The reference group is those who had concentrations not exceeding 50 μg/L regardless of the duration of exposure.

In this study, we assessed the risk of malnutrition among the women of reproductive age *i.e.*, 20–45 years at several concentrations of arsenic in drinking water in rural Bangladesh. The findings suggest an association between chronic arsenic exposure through drinking water and malnutrition. An overall 1.9 fold (95% CI = 1.1–3.6) increase in poor nutritional status was observed among the arsenic exposed women compared to women who were not exposed to arsenic more than 50 μg/L.

Risks were generally higher for those exposed to arsenic more than 100 μg/L. Excess risk was observed among the women who were exposed to arsenic more than 100 μg/L and for ≤10 years. Women exposed to arsenic more than 50 μg/L for more than 10 years showed weaker association. This may be due to inaccurate self reporting of arsenic exposure duration by the participants.

Similar observations have been reported from West Bengal, India and Taiwan. In an arsenic-exposed population in West Bengal body weight was negatively associated with the occurrence of keratosis [22] and in Taiwan vascular effects (blackfoot-disease) were associated with undernourishment (high intake of sweet potatoes, low intake of rice and vegetables) [39]. In Taiwan, a low serum β-carotene concentration was associated with a higher prevalence of arsenic-related skin-cancer [40] and ischemic heart disease [41].

It is not clear how the nutritional status of an individual influences the arsenic metabolism and toxicity process. Experimental studies in rabbits have shown that diets with low amount of methionine, choline or proteins reduced the urinary excretion of DMA, an end product of methylation [25]. Whether it is also applicable for humans is not known. Zinc has been suggested to influence arsenic toxicity [5] and mice fed a selenium-deficient diet showed a slower elimination of arsenic than selenium-sufficient mice [42]. Also, dietary selenium supplementation was found to prevent cytotoxic effects of arsenic mice *in vivo* [43]. Given that a deficiency in folate and vitamin B12 might lead to decreased levels of S-adenosylmethyltransferase [44], it may also result in decreased methylation of arsenic. Indeed, folic acid was found to protect mouse embryo fibroblasts against cytotoxicity of arsenic [45].

Chronic arsenic exposure has been reported to be associated with human adverse pregnancy outcomes in a few studies [11–14,16]. Animal model experimentation have also shown the toxicity of several forms of arsenic on pregnancy outcomes including malformations [46]. Recent studies reported that arsenic crosses the human placenta [47], although more than 90 percent of the arsenic in plasma and urine was in the form of dimethylarsenic acid indicating an increase in the methylation of arsenic during pregnancy. Maternal toxicity has also found to be associated with the adverse developmental effects of arsenic exposure. There are instances that the maternal toxicity is the causative factor in abnormal development of the embryos [48]. This is probably due to induction of metallothionein in the maternal liver that leads to a systemic redistribution of zinc and a transitory, but developmentally adverse zinc deficiency. These effects were produced in pregnant rat by arsenate. Exposure to arsenic also exerted direct adverse effects on explanted rodent embryos exposed to arsenic in the absence of the maternal system. However, a poor correlation was found on maternal and developmental toxicity relationship in an extensive literature analysis [49]. Therefore, arsenic is likely to have direct toxic effects on embryos *in vivo*, but its effects might be exacerbated by external toxicity [4]. Therefore, combined effects of malnutrition and chronic arsenic exposure may worsen the reproductive health of the women of particular age group.

To the best of our knowledge, this is the first study carried out to determine the effect of chronic arsenic exposure on the nutritional status of the women of reproductive age. None of the study participants had visible arsenical skin lesions. Therefore, the observed risk of malnutrition in this study is likely to be the effect of chronic arsenic exposure than to be the consequence of arsenical skin lesions.

We used BMI as the indicator of nutritional status in this study. Of the anthropometric indices, body mass index is considered to be more nutritionally than genetically related [50]. Thus it is appropriate to use BMI as an indicator of the adult nutritional status. However, body mass index is not the only indicator for measuring nutritional status. So, further studies including detail assessment of nutritional status together with evaluation of dietary intake and blood studies is required to understand the dynamics of malnutrition.

In case-control studies, exposure measurement remains as an important issue. In our study, we only collected water sample from one tube well for each participant’s exposure measurement. This single water measurement may not represent the historical arsenic exposure over time. However, due to absence of any reliable information on past exposure, it was essential to assume that arsenic concentrations from the tube wells had been relatively constant over time. The historical consistency of arsenic concentration is of particular concern with shallow ground water, which might be subject to greater fluctuation than water from a deeper well [5]. However, any fluctuation of arsenic concentrations is likely to affect the cases and controls equally. Interviewer bias is less likely because the interviewers were unaware of the arsenic concentration levels of the participant’s usual drinking water source. Nevertheless, strength of this study is the study design, availability of individual arsenic exposure data, BMI and determination of risk at different arsenic concentration levels.

## 4. Conclusions

The findings of this study add to the evidence that chronic arsenic exposure is likely to contribute to poor nutritional status among women of 20–45 years.

## Figures and Tables

**Table 1:** Baseline characteristics of the cases and controls *.

**Variable**	**Cases (n = 348)**	**Controls (n = 360)**	***p***
Age (years); mean ± sd	29.9 ± 7.8	30.4 ± 8.1	0.32
Occupation Housewife Others	94.8 5.2	92.5 7.5	0.23
Educational status No formal education Primary Secondary or above	24.7 43.1 32.2	23.2 47.2 29.6	0.16
Roof type Tin Others	94.5 5.5	94.7 5.3	0.27
Total monthly household income (US $); mean ± sd	47.3 ± 37.7	55.1 ± 46.5	0.02
Religion Muslim Others	74.7 25.3	68.3 31.7	0.06
Age at menstruation (years); mean ±_sd	12.6 ± 0.79	12.6 ± 0.79	0.46
Duration of menstruation (days); mean ±_sd	3.8 ± 1.4	3.7 ± 1.2	0.58
Age at marriage (years); mean ± sd	16.1 ± 2.2	16.1 ± 1.8	0.92
Total number of pregnancy/woman; mean ± sd	3.6 ± 2.4	3.6 ± 2.1	0.92
Arsenic concentration (μg/L); 0–50 >50–100 >100	24.1 15.6 60.3	36.4 14.7 48.9	<0.01
Duration of arsenic exposure (years); mean ± sd	7.8 ± 7.1	7.9 ± 6.4	0.78
Height (metres); mean ± sd	1.51 ± 0.05	1.51 ± 0.05	0.13
Weight (kilogram); mean ± sd	39.5 ± 3.4	47.9 ± 5.7	<0.01
Body Mass Index (BMI); mean ± sd	17.1 ± 0.9	20.9 ± 1.8	<0.01

* Results are expressed as percent, unless otherwise indicated.

Results regarding the association of arsenic concentration and nutritional status are given in Table 2.

An increased risk of malnutrition is observed for arsenic over 50 μg/L.

**Table 2:** Association between arsenic concentrations with nutritional status of the women of reproductive age*.

**Arsenic concentration (μg/L)**	**Cases (n = 348) (BMI < 18.5)**	**Controls (n = 360) (BMI > 18.5)**	**Odds ratio (OR)**	**95% CI**
≤50 ^†^	84	131	1.0	-
>50	264	229	1.9	1.1–3.6
>50–100 ^‡^	54	53	1.9	0.8–4.3
>100	210	176	2	1.1–3.6

* Ajusted for age, religion, participant’s education, monthly income and history of oral contraceptive pills.

‡ Adjuste for age, religion, monthly income and history of oral contraceptive pills.

† Referene category.

**Table 3:** Association between arsenic concentrations and duration of exposure with nutritional status of the women of reproductive age*.

**Duration of As exposure**	**As (in μg/L)**	Cases* (n = 342) (BMI < 18.5)	**Controls (n = 360) (BMI > 18.5)**	**Odds Ratio (OR)**	**95% CI**
Any duration ^†^	50	78	131	1	-
≤10 years	>50–100	44	38	1.9	0.7–5.5
	>100	156	132	2.2	1.1–4.3
>10 years	>50–100	10	15	1.9	0.7–5.2
	>100	54	44	1.7	0.8–3.3

* missing = 6.

* Adjusted for age, religion, type of roof, participant’s education, monthly income and history of oral contraceptive pills.

† Reference category.
